# Supplementary material for: On the design of random metasurface based devices
Source: Sci Rep. 2018 May 8;8:7162. doi: 10.1038/s41598-018-25488-4 (PMC5940824; doi:10.1038/s41598-018-25488-4)
Supplement: Supplementary file 1 — Supplementary Information: On the design of random metasurface based devices [file 41598_2018_25488_MOESM1_ESM.docx]

Supplementary Information: On the design of random metasurface based devices

Matthieu Dupré^1^, Liyi Hsu^1^, Boubacar Kanté^1^

UC San Diego, Department of Electrical and Computer Engineering, 9500 Gilman Drive, La Jolla CA 92093, USA

[bkante@ucsd.edu](mailto:bkante@ucsd.edu)

## Sensitivity of the elements to orthogonal polarization:


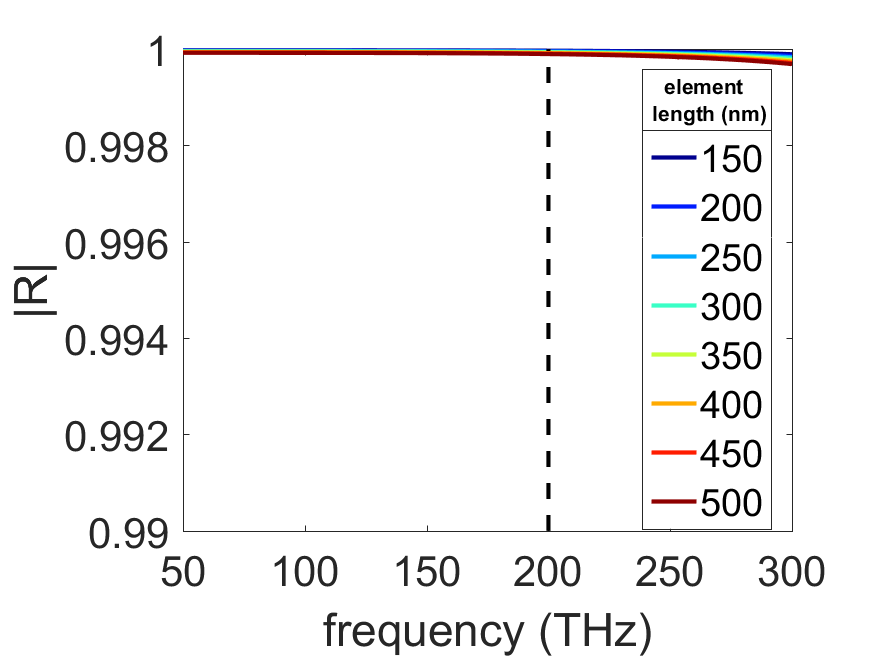

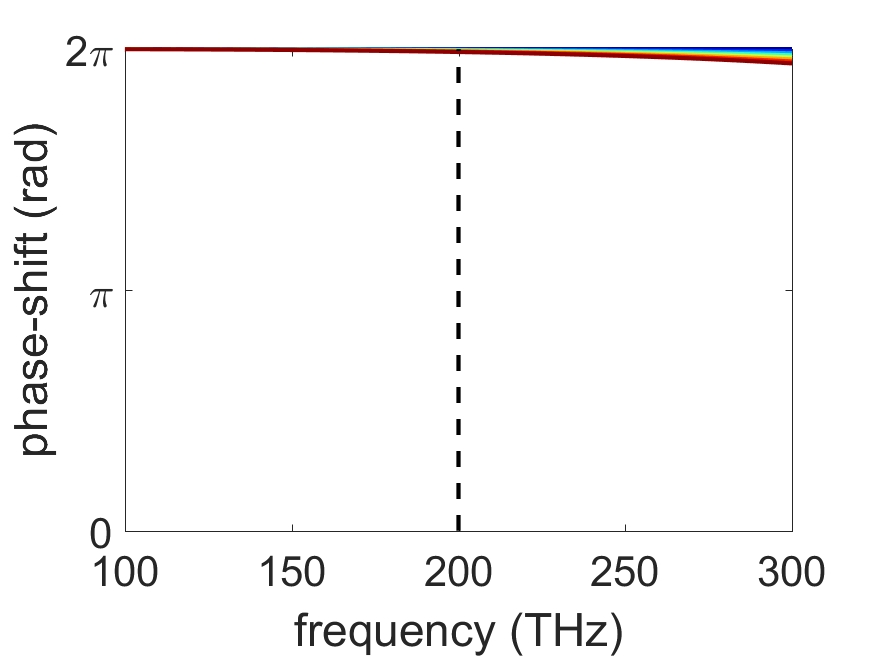


Figure S1 Magnitude and phase of the coefficient of reflection for an incident wave orthogonal to the longer dimension of the elements. Period is p_y_=100 nm, and spacer thickness is h_spacer_=70 nm.

## Phase shift of the periodic unit cell simulations vs spacer thickness


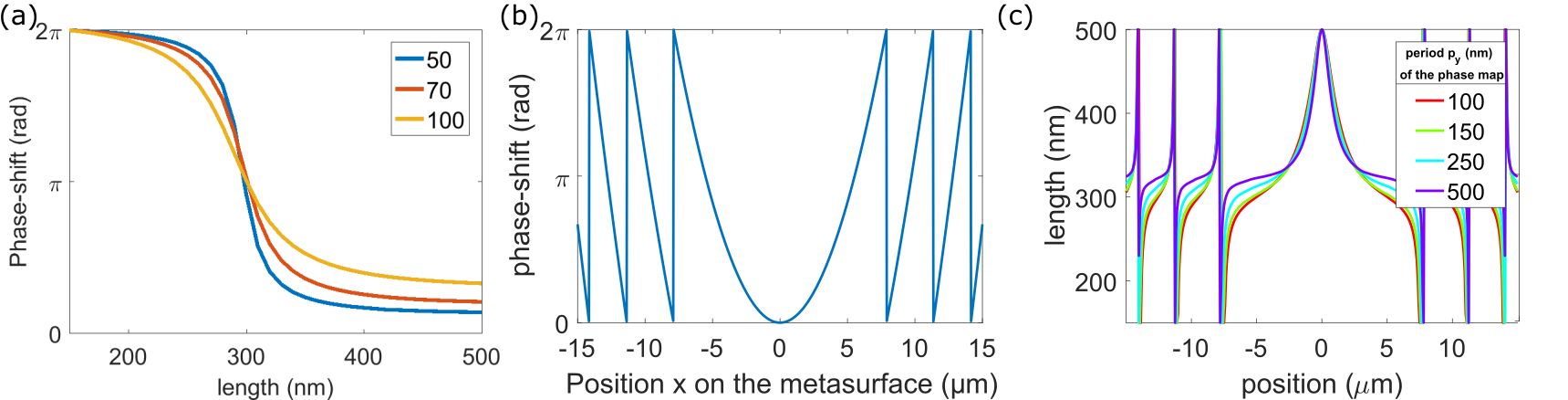


Figure S2 Phase-shift for different SU8 spacer thicknesses of 50 nm, 70 nm and 100 nm: Phase shift provided by an element at 200 THz as a function of the length of the elements.

## Effect of a ground plane made with a real metal (gold) on the reflection

In metasurface simulations, extensively used in the paper, meshing the layer of gold (ground plane) significantly increases our limited computational time on a node of the cluster by about 50% compared to the same simulation using perfect electric conductor (PEC) for the ground plane and Drude for the scatterers. In our simulations, we consider this metallic ground plane to be a PEC. However, the results are very similar with a real metal such as gold or silver. In the figures below, we plot the unit cell simulations (that are less memory intensive) of the reflection coefficients for a ground plane made of gold and simulated with a Drude model but with the scatterers always modeled with a Drude model. Compared with PEC ground plane, resonances and the phases are shifted to higher frequencies by about 20 THz which would shift the focusing wavelength of our metasurfaces to higher frequencies. To keep the same focusing frequency (200 THz, λ=1.5 μm), a simple workaround would consist in redesigning new reference phase-maps taking into account this gold mirror, which would basically come to decreasing the elements length by 30 nm. Under such condition we get similar results.


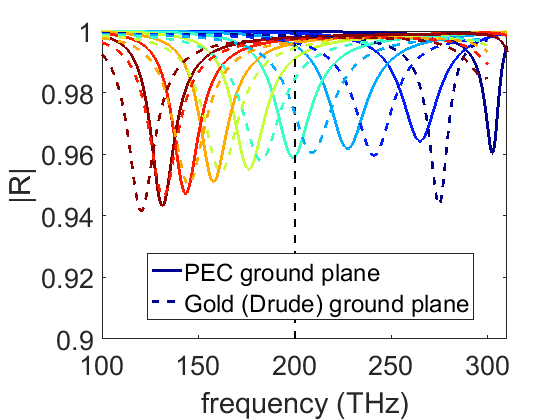

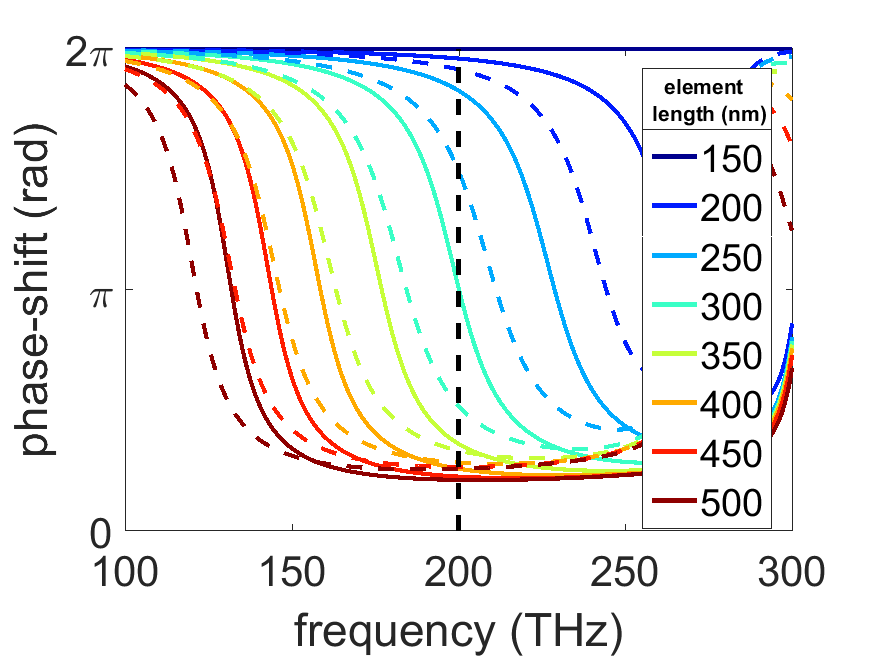


Figure S3 Magnitude (left figure) and phase (right figure) of the coefficient of reflection as a function of the frequency for different element lengths. Unit cell simulation for py = 100 nm and with a metallic ground plane modeled with Drude model for gold. Solid lines: PEC ground plane. Dash lines: gold ground plane modeled with Drude model.

## 1D periodic metasurfaces

We conduct a similar study than the 1D random metalenses but for periodic metalenses. In this case the density is directly related to the period of the metasurface with a density of 25/λ_0_^2^ corresponding to a period in y of 100 nm, a density of 16.7/λ_0_^2^ corresponding to a period of 150 nm etc. Periodic metasurfaces provide slightly better performance than random ones (+15%). The period in *x* direction is 900 nm. Hence the geometry matches very well with the one of unit cell simulations from which we extract the phase-maps, hence the improved performances compared to the random case. The total number of elements is the same than for the corresponding random metasurfaces and any other geometrical parameters are kept constant (width = 30 μm, focal length = 20 μm).


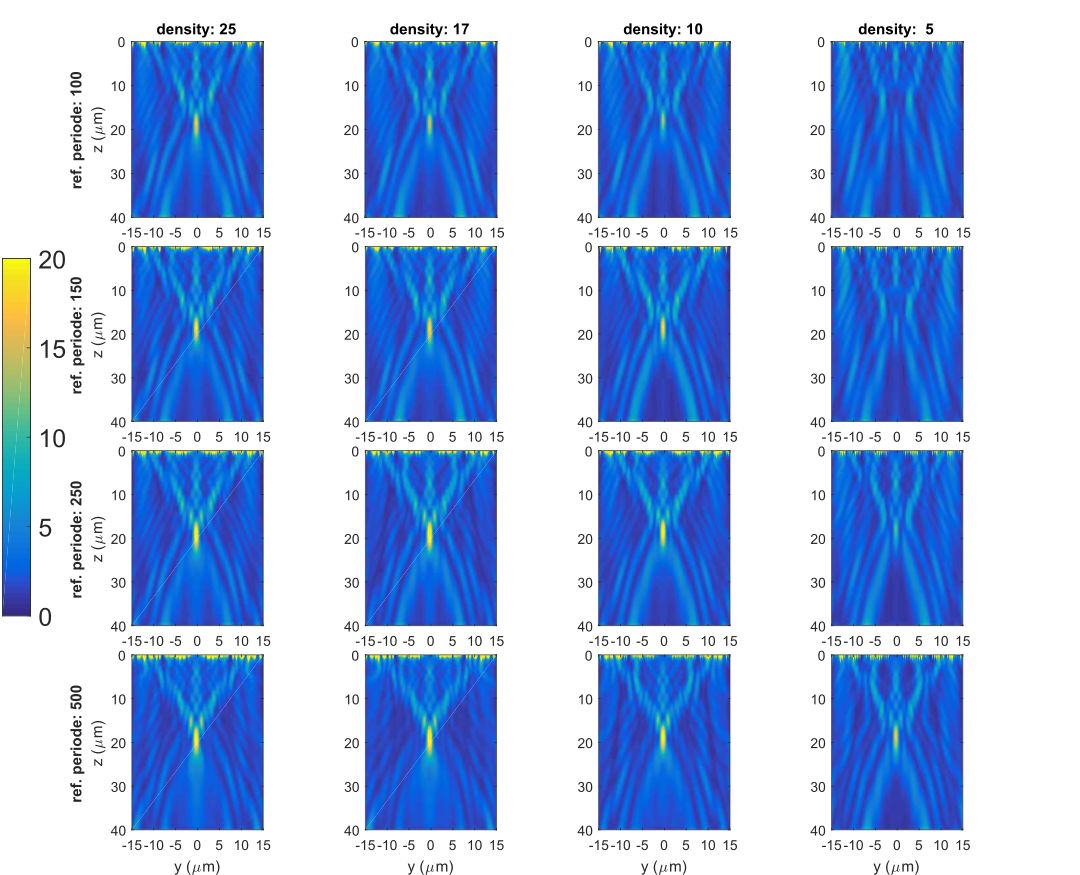


Figure S4. Density of energy of the 16 1D periodic metalenses. Designed for densities of 25/λ_0_^2^, 17/λ_0_^2^, 10/λ_0_^2^ and 5/λ_0_^2^ and for phase-maps of period 100 nm,150 nm, 250 nm and 500 nm.

## Standard deviation of the energy of 1D random metasurfaces

In the main article, we have shown the average results of 10 metasurfaces. Here, we present the standard deviation that is of the order of 10% of the energy of the field. This proves that random metasurfaces provide similar efficiencies, despite they small sizes.


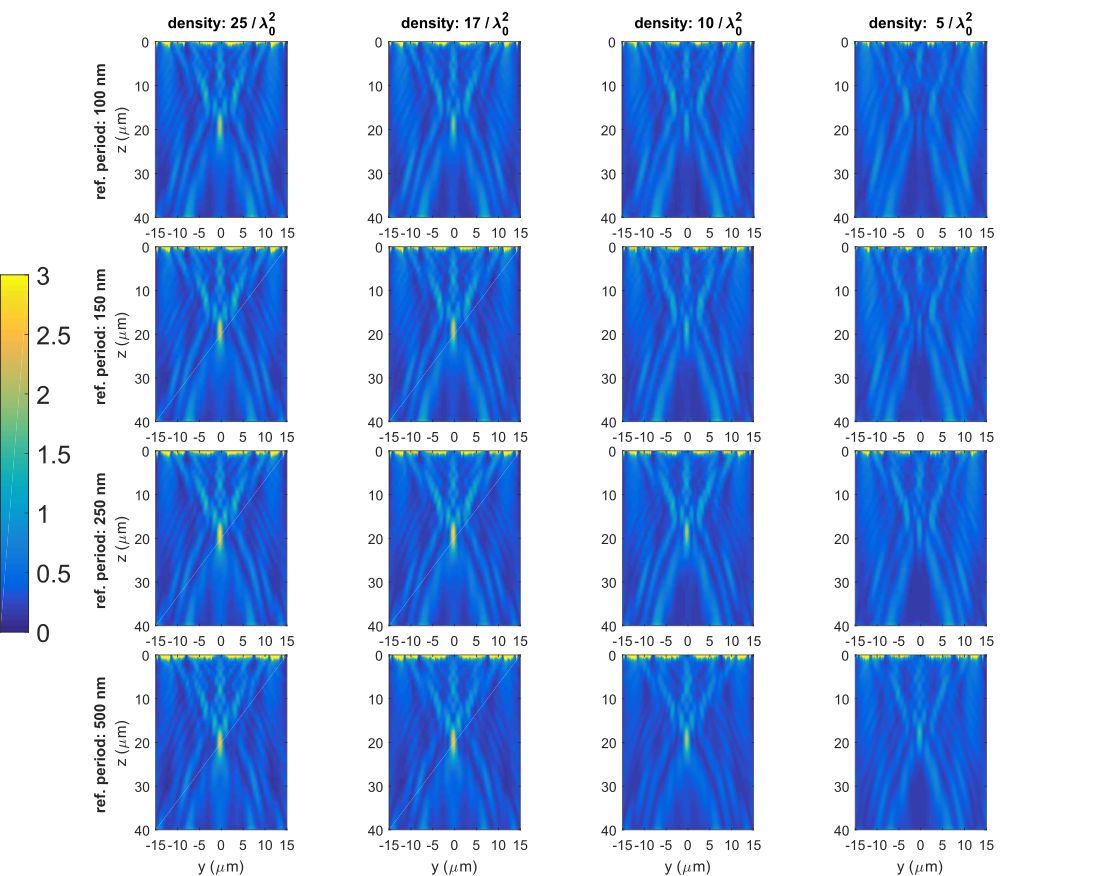


Figure S5. Standard deviation of the density of energy of the 16 1D random metasurfaces, computed on 10 samples.

## 2D periodic metasurfaces

Similar to the 1D case, we investigate periodic 2D metasurfaces. Those periodic metasurfaces have the same number of nano-resonators than the corresponding random metalenses, and the same geometrical parameters (width =10 μm, focal length = 10 μm), but in a periodic grid with *x* period of 900 nm and *y* period corresponding to 100 m, 150 nm, 250 nm, and 500 nm. Here, we can see that periodic metasurfaces provide a higher density of energy at the focal spot but only for a single polarization.


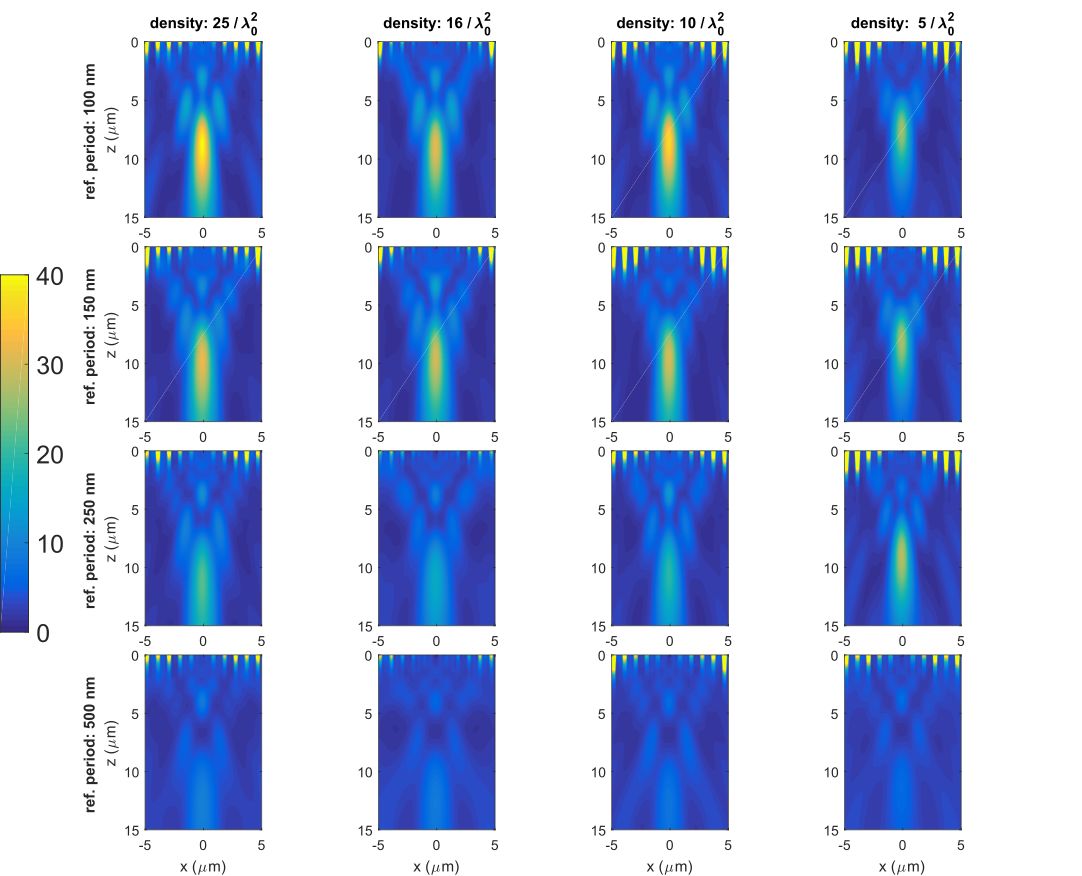


Figure S6. Density of energy of 2D random metasurfaces. Influence of the density of elements and reference phase-maps on the focusing of 2D random metalenses for sets of 16 metasurfaces designed with different densities and phase-map. The metasurfaces are located in the plane z=0.

## Standard deviation of the energy of 2D random metasurfaces

Here, we present the standard deviation of the energy for the 2D random metasurfaces. As in the 1D case, it is generally lower than 10% of the average value, expect for one metasurface, for which the standard deviation reaches about 15% of the value of the average energy in the vicinity of the focal spot. We can note that this higher standard deviation is achieved for a metasurface of quite low density, which means a lower number of elements, which makes it quite sensitive to randomness.


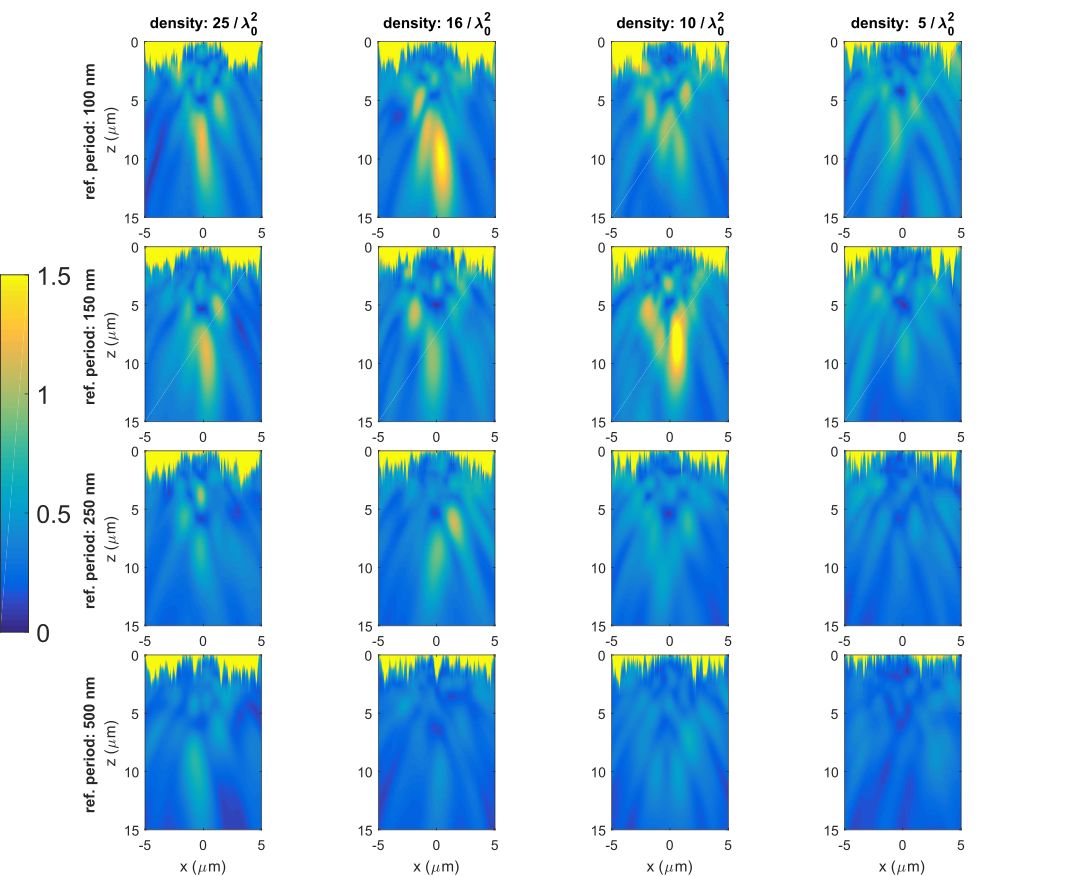


Figure S7. Standard deviation of the density of energy computed on 10 samples of 2D random metasurfaces. Influence of the density of elements and reference phase-maps on the focusing of 2D random metalenses for sets of 16 metasurfaces designed with different densities and phase-maps. The metasurfaces are located in the plane z=0.
